# Hyperparathyroidism Jaw Tumor Syndrome Presenting as Recurrent Femur Fractures in a Young Woman; a Rare Presentation of a Rare Disease

**DOI:** 10.1155/2020/9298147

**Published:** 2020-03-16

**Authors:** Piyumi S. A. Wijewickrama, Noel P. Somasundaram

**Affiliations:** Endocrinology Unit, National Hospital of Sri Lanka, Colombo, Sri Lanka

## Abstract

**Background:**

Primary hyperparathyroidism usually occurs secondary to parathyroid adenoma, multiglandular hyperplasia, or parathyroid carcinoma. The patients usually present with incidentally discovered high calcium level and systemic or skeletal manifestations. In young patients with primary hyperparathyroidism, familial syndromes including multiple endocrine neoplasia types 1, 2, and 4 and hyperparathyroidism jaw tumor syndrome should be considered. *Case Description*. We present a case of a 22-year-old Sri Lankan woman who presented with femur fractures in a background of childhood nephroblastoma and maxillary fibro-osseous tumor. The patient had biochemical parameters suggestive of primary hyperparathyroidism with a parathyroid mass. The histology following excision of the mass revealed a parathyroid adenoma. Based on the associated clinical manifestations, hyperparathyroidism jaw tumor syndrome was suspected, and genetic studies reported a positive CDC73 mutation with a whole-gene deletion of exon 1–17.

**Conclusion:**

Hyperparathyroidism jaw tumor syndrome is an important diagnosis to consider in a young patient presenting with classic clinical features due to the risk of malignancy, familial involvement, and need to monitor for progressive systemic manifestations. As this is a rare disease, it can often be missed due to low degree of suspicion and the ability of the jaw tumor to mimic a metastatic deposit.

## 1. Background

The most common presentation of primary hyperparathyroidism (PHPT) is asymptomatic hypercalcemia, which is incidentally detected in 80% of the patients [1]. PHPT was first described about 90 years ago when the patients presented with classic symptoms depicted by “bones, stones, abdominal groans, and psychic moans,” describing skeletal manifestations, nephrolithiasis, constipation and abdominal pain, and confusion [2]. While nephrolithiasis and skeletal manifestations are a direct result of excess parathyroid hormone (PTH) levels, anorexia, nausea, constipation, polydipsia, and polyuria are caused by hypercalcemia [3].

The skeletal manifestations are mainly due to reduced bone mineral density (BMD), which is more remarkable in the cortical bones such as forearm and hip than in the trabecular bones. The risk of hip fractures due to primary hyperparathyroidism has contrasting evidence. A large population-based cohort study carried out in Sweden with 19 years of follow-up revealed an increased risk of hip fractures in men but not in women [4]. Another population-based study showed a significantly increase in risks of vertebral, Colles', rib, and pelvic fractures in PHPT patients, but proximal femur fractures showed only a marginal rise in these patients, with female gender and increasing age identified as risk factors [5].

PHPT occurs mainly due to benign parathyroid adenoma in 80%, while multiglandular parathyroid hyperplasia contributing to 15–20% and parathyroid carcinoma to 0.5% [3]. The majority of PHPT patients present at the age of 55 to 60 years, and incidence among females is twice that of males [6]. In a young patient presenting with PHPT, familial syndromes including multiple endocrine neoplasia (MEN 1), MEN 2, MEN 4, and hyperparathyroidism jaw tumor syndrome (HPT-JT), familial isolated hyperparathyroidism should be looked for, which contribute to around 10% of PHPT cases [7].

HPT-JT is an autosomal dominant syndrome with incomplete penetrance. The patients present with parathyroid tumors, ossifying fibromas of jaw, and a variety of renal and uterine tumors [8, 9]. This is due to mutation of CDC73 tumor suppression gene, which encodes the protein named Parafibromin, which is responsible for the cell cycle arrest [8]. This is an exceedingly rare syndrome where a total of about 100 index cases of CDC73 mutation carriers have been identified to date, without a clear correlation between genotype and phenotype [7]. HPT-JT patients present with parathyroid carcinoma or adenoma at an earlier age of onset with a mean age of 33 years, and they are particularly at a higher risk of developing parathyroid carcinoma.

We report a case of a young female presented with recurrent low trauma femur fractures, with a significant past history of Wilms' tumor and maxillary tumor, who was ultimately diagnosed to have HPT-JT syndrome.

## 2. Case Description

**Figure 1:**
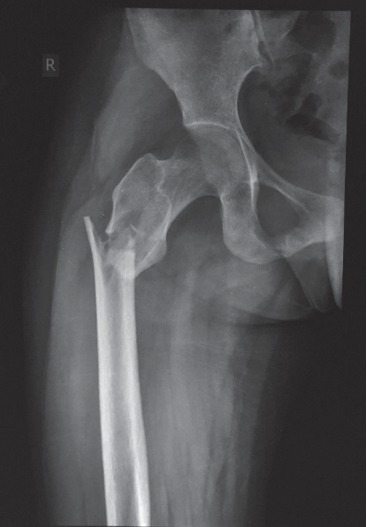
X-ray showing the right subtrochanteric fracture.

A 22-year-old Sri Lankan woman was referred from the orthopedics unit to the endocrinology unit, National Hospital of Sri Lanka, for further evaluation of recurrent femur fractures. She first experienced a right sided subtrochanteric fracture following a low trauma injury at the age of 20 years (Figure 1), for which she underwent internal fixation. Thereafter, she experienced a left intertrochanteric femur fracture at the age of 22 years, 2 months prior to the current presentation.

She had an interesting past medical history. She was diagnosed to have a left side nephroblastoma at the age of 8 years, for which she underwent left side radical nephrectomy followed by chemotherapy. Thereafter, at the age of 14 years, she developed a right sided facial swelling and was found to have a maxillary tumor which was described during the surgery as a well-circumscribed mass lesion in the left maxilla, eroding the anterior wall of maxilla and lateral nasal wall. She underwent partial maxillectomy for this, and histology revealed features suggestive of fibrous dysplasia.

She did not have a history of chronic steroid use, hypogonadism, hyperthyroidism, rheumatoid arthritis, and exposure to cigarette smoking to suggest secondary causes for osteoporosis. She had neither any features of malabsorption nor inadequate exposure to sunlight to suggest a vitamin D deficiency causing these fractures. The histology of bone biopsies was negative for metastatic deposits.

She did not have a family history of hyperparathyroidism, renal, or uterine tumors. There was no past history or family history suggestive of MEN 1. Her general and systemic examinations were normal apart from the healing fracture sites.

Her investigation findings are summarized in Table 1.

Her investigations revealed high total calcium and urinary calcium excretion, with low serum phosphate level. She was found to have a vitamin D deficiency, which was corrected by vitamin D replacement. As high calcium and low phosphate were suggestive of a primary hyperparathyroidism, intact parathyroid hormone (PTH) was done after correcting vitamin D level, which was found to be very high at 1025 pg/ml (10–65 pg/ml), confirming the diagnosis of hyperparathyroidism.

Her left forearm dexa scan revealed osteoporosis with a *Z* score of −5.6, suggestive of active chronic hyperparathyroidism, leading to bone resorption and reduced bone mineral density with preferential involvement of cortical bone and increased risk of fracture [10].

**Figure 2:**
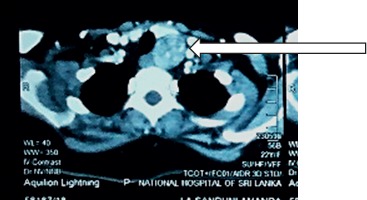
CT neck showing the left side parathyroid mass.

Three-dimensional computed tomography (CT) of neck was carried out for localization, which revealed a 2.5 × 2.9 cm well-defined rounded mass with avid contrast enhancement posteroinferior to the left lobe of the thyroid gland, suggestive of a parathyroid mass (Figure 2). Nuclear imaging was not done as the facility was not available at our setting.

At this point, due to the constellation of nephroblastoma, maxillary tumor, and primary hyperparathyroidism with a parathyroid mass, a possibility of hyperparathyroidism jaw tumor syndrome was suspected although a suggestive family history was not there. Therefore, the genetic studies were carried out via target region capture followed by next generation sequencing, using a specific indel detection tool, which revealed a positive CDC73 mutation with a large deletion of exon 1–17. This mutation was verified by reverse transcription polymerase chain reaction (RT-PCR), where exons 1, 10, and 17 were selected for verification. This confirmed the diagnosis of hyperparathyroidism jaw tumor syndrome (Table 2). MEN 1 and calcium sensitive receptor mutations were negative.

**Figure 3:**
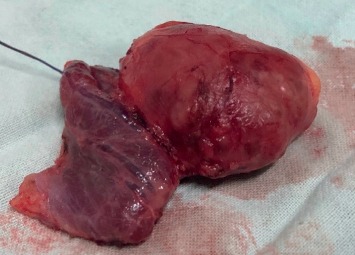
Macroscopic appearance of the excised parathyroid mass with hemithyroidectomy.

The patient underwent subtotal thyroidectomy with parathyroidectomy with removal of left lower parathyroid mass, which was a smooth, round, well-demarcated mass (Figure 3), and the remaining parathyroid glands were atrophied.

Immediate postoperative PTH level was reduced by more than 50%–76 pg/ml, suggesting successful removal of the parathyroid mass. The histology revealed encapsulated parathyroid adenoma composed of a proliferation of polygonal cells separated by fibrovascular septa, with round to ovoid nuclei with dispersed chromatin and inconspicuous nucleoli and moderate eosinophilic cytoplasm. Mitoses were rare, and there were also cystic areas. There was no evidence of vascular invasion, necrosis, or invasion into soft tissues of thyroid gland. These features were compatible with a parathyroid adenoma.

Currently, the patient is on calcium and calcitriol treatment and being followed up with serum calcium and phosphate levels with monitoring of urinary calcium excretion. Her imaging of the abdomen did not reveal a recurrence of nephroblastoma or uterine tumors. Biochemical screening of family members was negative, and genetic screening is underway.

## 3. Discussion

This case depicts a rare presentation of a rare disease. The typical initial presentation of individuals with HPT-JT is reported to be jaw and teeth deformities as a result of ossifying fibromas of mandible or maxilla [9]. In contrast, this patient's initial clinical manifestation was a nephroblastoma developed during childhood. Renal manifestations, including renal cysts, hamartoma, and nephroblastoma (Wilms' tumor), are well described but rare manifestations of HPT-JT syndrome. Wilms' tumors have been described in less than 3% of patients with the mutation, and despite a through literature survey, we did not come across cases where nephroblastoma has presented as the initial manifestation of HPT-JT syndrome as in this case [9, 11, 12].

Ossifying fibromas of the jaw, which this patient developed as the second manifestation of the disease at the age of 14 years, is a well-described manifestation of HPT-JT syndrome, which is shown to occur in 50% of these patients, usually in the adolescent age [9, 11]. These are benign lesions which demonstrate locally invasive features. The distinction from brown tumors should be done histologically, and these lesions reveal features of fibrous dysplasia as seen in this patient. In this patient, as the maxillary tumor developed following the nephroblastoma, malignant metastatic deposit was suspected, which was excluded by histology. This sequence of clinical manifestations may have led to a delay in diagnosis as well.

The primary manifestation of hyperparathyroidism in this patient was bilateral low trauma femur fractures, which could have been contributed by the coexisting vitamin D deficiency. Vitamin D deficiency is known to be more prevalent among patients with primary hyperparathyroidism, due to multiple possible underlying mechanisms, such as increased conversion of 25-hydroxy vitamin D to 1,25-dihydroxy vitamin D and increased hepatic inactivation of 25-hydroxy vitamin D level [2, 13]. Therefore, patients with coexistent severe vitamin D deficiency usually have more biochemically and clinically severe PHPT as seen in our patient. Although parathyroid carcinomas have been reported relatively frequently in HPT-JT patients, our patient's histology was suggestive of a parathyroid adenoma. As HPT-JT syndrome patients are known to be susceptible for parathyroid carcinoma as well as high rate of recurrence of parathyroid disease, this patient warrants a close follow-up with frequent monitoring [14].

With regard to the types of CDC73 mutations reported in the literature, the majority of CDC73 mutations are frameshift, nonsense, and missense variants, as well as small deletions and insertions [7]. In contrast, our patient had a whole-gene deletion of exons 1–17 of CDC73 gene, which is reported infrequently in the literature [14–16]. In fact, gross deletions are reported only in 1% of the cases of CDC73 mutations, which makes this case more significant [15, 17].

Our patient's atypical sequence of clinical manifestations with initial development being a nephroblastoma prompted the clinicians to suspect metastatic deposits at each of subsequent developments of maxillary tumors and femur fractures, deviating them from considering this familial syndrome for several years, which contributed to a delay in diagnosis of the condition.

## 4. Conclusion

HPT-JT syndrome, although rare, is an important diagnosis to consider, especially in young patients presenting with classical constellation of clinical manifestations of parathyroid tumor, maxillary or mandibular tumors, and renal or uterine tumors, due to its risk of malignancy and importance of family screening. It needs close monitoring for new developments. The above case highlights an atypical presentation of this rare disease, which presented with nephroblastoma as the first clinical manifestations. Furthermore, this case is significant as the patient carries a rare type of mutation of a whole-gene deletion from exons 1–17 of CDC73 gene. To the best of our knowledge, this is the first genetically confirmed HPT-JT syndrome case reported from Sri Lanka.

## Figures and Tables

**Table 1:** Summary of investigations.

Investigations	Results	Normal range
Total calcium	3.57 mmol/l	(2.2–2.7)
Serum phosphate	0.55 mmol/l	(1.12–1.45)
Urine calcium excretion	10.8 mmol/l/24 h	(2.5–6.26)
Urine phosphate excretion	21.42 mmol/l/24 h	(12.9–4.2)
Serum alkaline phosphatase level	1633 *μ*/l	
Serum 25(OH) vitamin D level	7.51 *μ*/l	
Thyroid stimulating hormone	1.85 mIU/l	(0.4–4)
Free T4 level	0.99 ng/dl	(0.7–1.9)

**Table 2:** Characteristics of positive CDC73 mutation.

Gene	Nucleic acid alteration	Mutation location	Type
CDC73	EX 1–17 DEL	EX 1–17/CDS 1–17	Heterozygous
